# Diverse plasma membrane protrusions act as platforms for extracellular vesicle shedding

**DOI:** 10.1002/jev2.12148

**Published:** 2021-09-17

**Authors:** Kirsi Rilla

**Affiliations:** ^1^ Institute of Biomedicine University of Eastern Finland Kuopio Finland

**Keywords:** actin, extracellular vesicle, filopodium, microvillus, shedding

## Abstract

Plasma membrane curvature is an important factor in the regulation of cellular phenotype and is critical for various cellular activities including the shedding of extracellular vesicles (EV). One of the most striking morphological features of cells is different plasma membrane‐covered extensions supported by actin core such as filopodia and microvilli. Despite the various functions of these extensions are partially unexplained, they are known to facilitate many crucial cellular functions such as migration, adhesion, absorption, and secretion. Due to the rapid increase in the research activity of EVs, there is raising evidence that one of the general features of cellular plasma membrane protrusions is to act as specialized platforms for the budding of EVs. This review will focus on early observations and recent findings supporting this hypothesis, discuss the putative budding and shedding mechanisms of protrusion‐derived EVs and their biological significance.

**Abbreviations**
AFM: atomic force microscopy
CLEM: correlative light and electron microscopy.
ECM: extracellular matrix
EV: extracellular vesicle
HA: hyaluronan
HAS: hyaluronan synthase
MSC: mesenchymal stem cell

## 1 DIVERSITY OF EXTRACELLULAR VESICLES (EV)

Extracellular vesicles (EV) are nanoscale cell fragments shedding from all cell types and released into the extracellular environment and body fluids. They are covered with lipid bilayer membrane and carry a multitude of molecules such as proteins, signaling molecules (Choi et al., 2019), carbohydrates (Gerlach & Griffin, 2016; Williams et al., 2018), and nucleic acids (Valadi et al., 2007) originating from the donor cell. Their capability to transfer these molecules to target cells represents a novel communication system between neighbouring and distant cells and tissues. Additionally, these unique properties of EVs provide huge future possibilities for their utilization in clinical applications, such a diagnostics and carriers of therapy. However, to learn and take the whole advantage of these multipurpose medical tools, it is important to fully understand their biology and properties. One of the central questions is to understand their release mechanisms.

**FIGURE 1:**
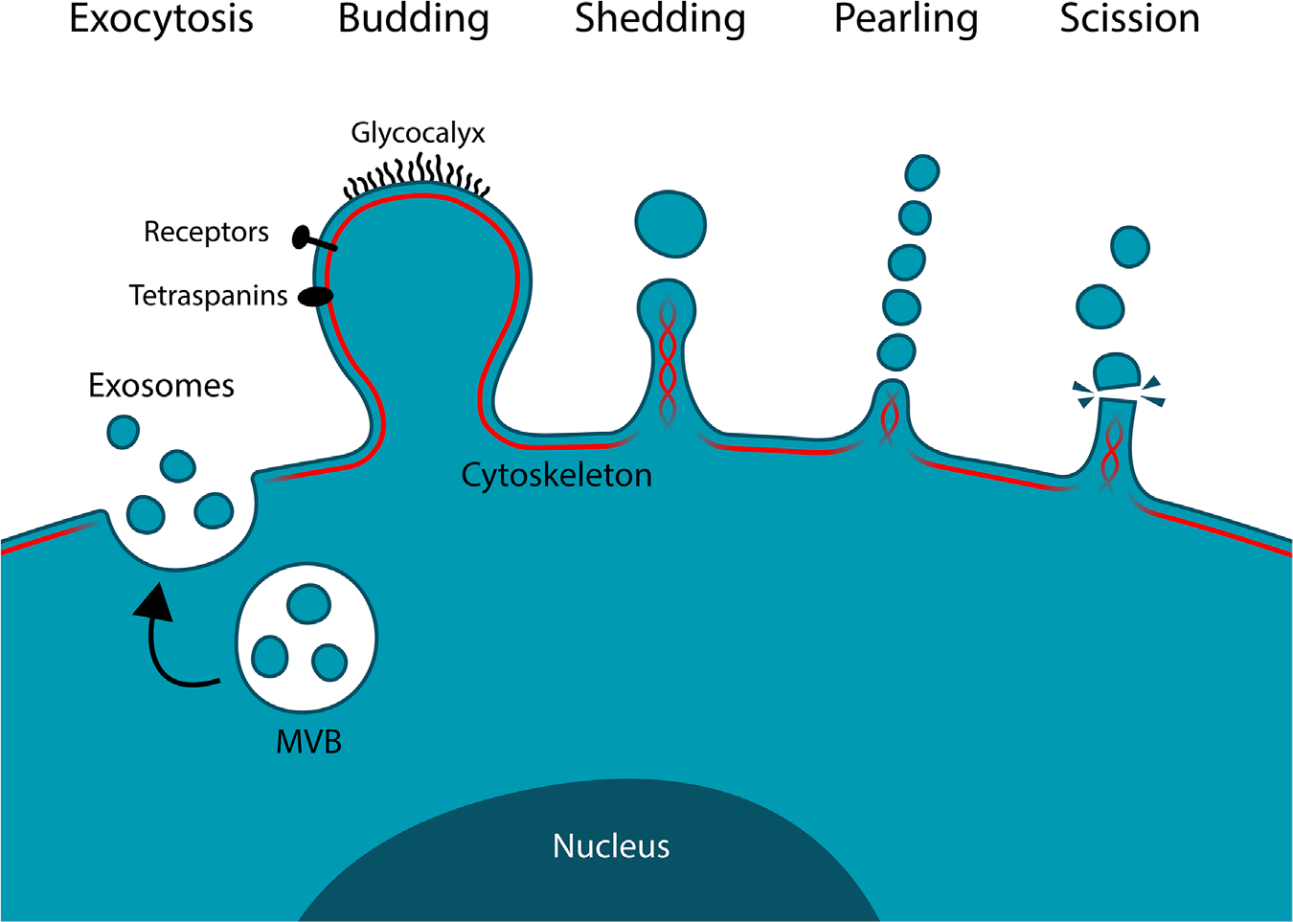
Origins of different classes of extracellular vesicles. Exosomes are formed by exocytosis of multivesicular bodies of endosomal origin and microvesicles or ectosomes are formed by the outward blebbing, shedding, pearling or scission of the plasma membrane or its various protrusions

EV is a generic term for several groups of cell‐derived particles that are generated through various cellular processes from various cell types. This diversity of EV has recently gained much attention (Minciacchi, et al. 2015), and according to the current understanding EV are shedding by several mechanisms (Figure 1). The broad term EVs is categorized into three major classes of lipid vesicles: exosomes, microvesicles and apoptotic bodies. This classification is based on the biogenesis of vesicles and on their difference in diameter. Exosomes are the smallest EVs, with a diameter ranging from 50 to 150 nm and are exocytosed from intracellular vesicles of endosomal origin called multivesicular bodies (MVBs). Microvesicles or ectosomes, due to their shedding from outer membranes, result by direct budding of the plasma membrane and their diameter ranges between 50 and 1000 nm. Apoptotic bodies are a product of apoptosis and contain the biomaterial from dying cells. The diameter of apoptotic bodies ranges from 50 to 5000 nm (Mathieu et al., 2019; Raposo & Stoorvogel, 2013).

To maintain simplicity, the mechanism of plasma membrane‐derived EV shedding is generally described as a result of budding from the plasma membrane of the main cell body. However, plasma membranes are dynamic, fluidic, and flexible structures and most cell types express variable extensions or protrusions on their plasma membranes, supported by cytoskeleton (Chhabra & Higgs, 2007). Interestingly, plenty of early findings show shedding of vesicles from plasma membrane protrusions of a variety of epithelial cell types (Beaudoin & Grondin, 1991) and there is growing evidence that different plasma membrane extension such as filopodia, microvilli, cilia and nanotubes act as a substantial source for EVs (Mathieu et al., 2019). This review article summarizes the data that support this mechanism of EV shedding and discusses the overall biological significance of cellular protrusions as sources of EV shedding in relation to EV originating from plain plasma membranes or via exocytosis. According to their budding and shedding from the outer plasma membranes, these EVs could be categorized into subpopulation of microvesicles. However, to avoid possible confusion, we will use the general term EV in this review article.

## 2 EVS ARE SHEDDING FROM PLASMA MEMBRANE EXTENSIONS OF MANY DIFFERENT CELL TYPES

Interestingly, several early findings that are currently considered as the very first indications of the existence of EVs, suggest the role of plasma membrane protrusions in the formation of EV. The first reported transmission electron microscopic visualization of EV derived from platelets by Peter Wolf, called ‘platelet dust’ (Wolf, 1967) and later publication by Polasek (Polasek, 1982) actually describe fractions originating from plasma membrane protrusions of platelets. Additionally, long slender pseudopods of erythrocytes were described to act as sources of vesicles (White, 1974). One of the first pieces of evidence of EV originating from cellular protrusion is placental cytotrophoblast microvilli that were shown to release cellular fragments (Enders, 1965). This was later supported by the finding that placental syncytiotrophoblast microvilli act as sources for EV (Van Der Post et al., 2011).

The existence of matrix vesicles, a specific population of EVs, derived from the plasma membrane of mineral forming cells of cartilage, bone, and dentin has been recognized for decades. Hale and Wuthier described that cytoplasmic processes of chondrocytes are the precursors of matrix vesicles and actin dynamics is essential for their formation (Hale & Wuthier, 1987). Correspondingly, Thouverey et al have shown that matrix vesicles originate from apical membrane microvilli of mineralizing osteoblast‐like Saos‐2 cells (Thouverey et al., 2009).

Several other findings in many different cell types support the essential role of plasma membrane extensions in EV release. One example is melanocytes that utilize shedding vesicles originating from filopodia (Scott, 2012) to transfer melanosomes to keratinocytes (Ando et al., 2012). Podocytes, a specific cell type covering the glomerular basement membrane in the kidney, release membrane vesicles into urine originating from tip vesiculation of long finger‐like microvilli (Hara et al., 2010). Additionally, microvilli of neuroepithelial cells (Marzesco et al., 2005) and cholesterol‐depleted colon carcinoma cells (Marzesco et al., 2009) release EVs. Furthermore, enterocyte microvillar tips generate vesicles, enriched in intestinal alkaline phosphatase, and deploying catalytic activity into the intestinal lumen (Mcconnell et al., 2009). Recent findings by Lai et al suggest EV shedding from tips of protrusions in membrane‐labelled (PalmGFP) human embryonic kidney 293T cells (Lai et al., 2015). There is also evidence on EV originating from cilia, for example from cilia of neuroepithelial cells (Dubreuil et al., 2007) and tracheobronchial epithelium (Kesimer et al., 2009).

**FIGURE 2:**
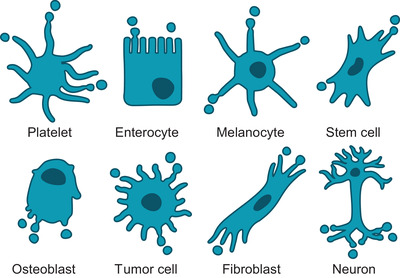
Examples of different cell types that generate EVs from their diverse plasma membrane protrusions and extensions

Cancer cells have typically abundant filopodia, which suggests that a substantial population of EV in cancer cell‐derived EV preparations is derived from filopodia. This has been shown in human chondrosarcoma, HAS3 overexpressing MCF7 breast cancer (Rilla et al., 2013), melanoma cells (Arasu et al., 2019; Deen et al., 2016), and HAS3 overexpressing MCF10A cells (Noble et al., 2020). Atomic force microscopy (AFM) and scanning electron microscopy (SEM) have shown that progesterone or PDGF‐BB treatments increase plasma membrane protrusions on the surface of adipose tissue‐derived mesenchymal stem cells (MSCs) (Casado et al., 2017). This suggests a connection between protrusions and EVs since these treatments also stimulate the secretion of EVs in MSCs. Examples of cell types that are suggested to release EVs from their diverse plasma membrane extensions are illustrated in (Figure 2) and summarized in (Table 1).

**TABLE 1:** Examples of various cell types and reported EV shedding from their diverse plasma membrane protrusions

Cell type	Protrusion described	Detection method(s)	Suggested/specific function	Reference
Platelet	Plasma membrane protrusions	TEM	Unknown	(Wolf, 1967)
Erythrocyte	Pseudopods	TEM	Unknown	(White, 1974)
Platelet	Platelet pseudopodium	SEM	Platelet activation	(Polasek, 1982)
Chondrocyte	Microvilli	TEM	Matrix vesicles	(Hale & Wuthier, 1987)
Chondrocyte	Cytoplasmic processes	TEM	Matrix vesicles	(Takagi et al., 1989)
Neuroepithelial cells	Microvilli	TEM, SEM	Brain development	(Marzesco et al., 2005)
Caco‐2 colon carcinoma	Microvilli	TEM	Differentiation	(Marzesco et al., 2009)
Enterocyte	Microvilli	TEM, SEM	Catalytic activity	(Mcconnell et al., 2009)
Osteoblast	Microvilli	TEM	Matrix vesicles	(Thouverey et al., 2009)
Tracheobronchial epithelial cell	Cilia	TEM	Mucosal defense	(Kesimer et al., 2009)
Megakaryocyte	Micropodia	TEM, DIC	Thrombosis	(Flaumenhaft et al., 2009)
Glomerular podocyte	Microvilli	TEM	Urine biomarker	(Hara et al., 2010)
Melanocyte	Dendrites	TEM, SEM	Transfer of melanosomes	(Ando et al., 2012)
Several cell types	Microvilli/filopodia	LSM, TEM	HAS3‐induced EV shedding	(Rilla et al., 2013)
Platelet	Flow‐induced protrusions	TEM, LSM	Proinflammatory function	(Tersteeg et al., 2014)
Normal rat kidney (NRK) cell	Retraction fibres	LSM	Migrasomes	(Ma et al., 2015)
Human embryonic kidney 293T cell	Tips of cell projections	LSM	Intercellular communication	(Lai et al., 2015)
Syncytiotrophoplast	Microvilli	TEM	Marker for preeclampsia	(Han et al., 2016)
MCF‐7 breast cancer cell	TNT	LSM, Phase contrast	Intercellular communication	(Patheja & Sahu, 2017)
Adipose tissue derived MSC	Plasma membrane protrusions	AFM, SEM	Unknown	(Casado et al., 2017)
Bone marrow derived MSC	Filopodia/retraction fibres	SEM, LSM	Unknown	(Arasu et al., 2017)
HT1080 fibrosarcoma	Retraction fibres	LSM, SEM (CLEM)	Adhesive exosome trails	(Sung et al., 2020)

Abbreviations: AFM, atomic force microscopy; CLEM, correlative light and electron microscopy; DIC, differential interference contrast microscopy; LSM, laser scanning microscopy; MSC, mesenchymal stem cell; SEM, scanning electron microscopy; TEM, transmission electron microscopy.

## 3 FILOPODIA AND OTHER ACTIN‐BASED PLASMA MEMBRANE PROTRUSIONS AS PLATFORMS FOR EV SHEDDING

The plasticity of the plasma membrane and a dynamic cytoskeleton are crucial for the formation of diverse finger‐like plasma membrane protrusions (Chhabra & Higgs, 2007). Because of their diverse morphology and existence of different protrusions in the same cell types or even the same individual cells in different circumstances, their nomenclature is often variable and inconsistent. The most extensively studied and described membrane extensions are filopodia (dorsal and lateral), tunnelling nanotubes, microvilli, retraction fibres, lamellipodia, invadopodia, ruffles and blebs (Chhabra & Higgs, 2007).

A complex intracellular molecular network of signaling factors and other molecules regulates the formation of cellular extensions. A key set of proteins drives the formation of filopodia; however, the relative importance of each protein seems to vary between cell types. Regulated assembly of the actin cytoskeleton, especially at filopodial tips, controls their extension and retraction (Mallavarapu & Mitchison, 1999), and the most important molecular mechanisms regulating this assembly include motor protein myosins, small GTPases of the Rho family, formins, and fascins (Mattila & Lappalainen, 2008). Membrane deformation during protrusion formation is driven by I‐BAR domain‐containing proteins such as IRSp53 (Ahmed et al., 2010).

The various functions of filopodia are widely known: They act as tentacles that cells use to probe and sense their microenvironment, as pioneers during cell invasion in the tight filamentous network of the ECM (Mattila & Lappalainen, 2008), and as mediators of adhesion (Jacquemet et al., 2015). Additionally, they are crucial precursors for dendritic spines in neurons (Sekino et al., 2007), osmotic sensors that resist and protect cells from osmotic stress (Karlsson et al., 2013), and important regulators of signaling during embryonic development (Pröls et al., 2016; Sagar et al., 2015). Filopodia are defined as dynamic protrusions adhered to substratum or another cell, while microvilli are more stable and not adhered, and typical for intestinal and kidney epithelial cells to increase the absorptive surface area (Chhabra & Higgs, 2007).

In addition to filopodia and microvilli, retraction fibres, vesiculation of protrusions (Sung et al., 2015, 2020), and reticular adhesions formed during mitosis (Lock et al., 2018) are substantial platforms of EV biogenesis. The release of EVs is a universally conserved process that occurs in many organisms. Interestingly, EVs originate from nanotubes or other extensions of archaea and bacteria, that resemble tunnelling nanotubes of eukaryotes (Gill et al., 2018). Tunnelling nanotubes are long, thin protrusions as a mechanism used by tumour cells to adhere to and migrate through the extracellular matrix (Wolf et al., 2020).

**FIGURE 3:**
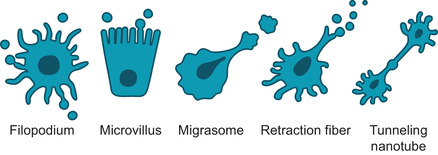
A schematic illustration of different plasma membrane protrusions that potentially act as platforms for the shedding of EVs. Protrusion‐derived EVs are generated by shedding from tips of filopodia or microvilli, from migrating cells as migrasomes, by pearling/vesiculation of retraction fibres, or via nanotubes

**FIGURE 4:**
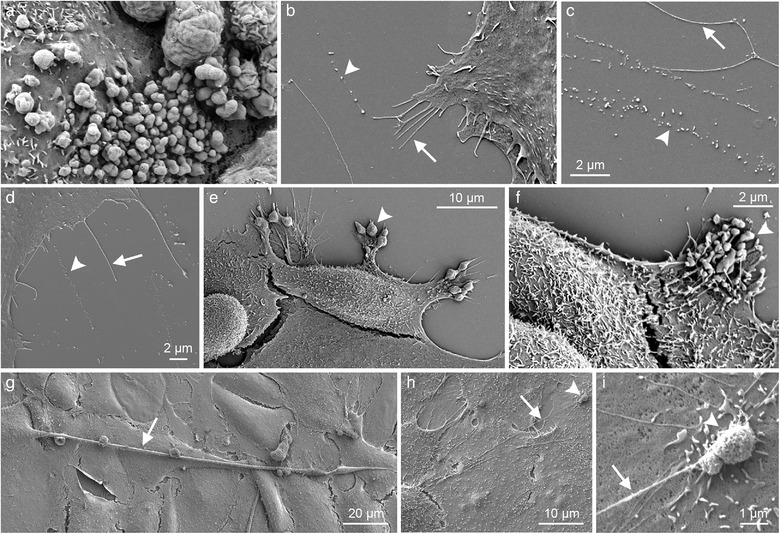
Scanning electron microscopy of cultured cells that release EVs from different protrusions: Plasma membrane budding (a), shedding or pearling of filopodia and retraction fibres (b‐d), migrasomes of migrating cells (e, f), and tunneling nanotubes (g‐i). MKN74 gastric cancer cells are shown in a, e, and f, human mesenchymal stem cells in b‐d, and primary mesothelial cells in g‐i. Arrows point filopodia and other protrusions and arrowheads indicate EVs of variable size in all panels. A big vesicle on the tip of a long tunneling nanotube from panel h is shown in the panel i at higher magnification (arrowhead in h and i)

Despite the clear indication of filopodia and other protrusions as platforms of EV shedding, this mechanism has received relatively little attention in recent publications. With the increasing interest and research activity on EVs, there is, however, growing evidence that cellular extensions act as specific sites for EV formation. This phenomenon provides a novel function for protrusions but is also potentially associated with many well‐described protrusion‐related functions such as migration, mediation of signaling, sensing environment, interaction with the extracellular matrix, and changing information between cells. All above‐mentioned observations support the hypothesis that EV release from different membranous extensions is a universal process, summarized in (Figure 3 and Figure 4).

## 4 ROLE OF ACTIN AND OTHER PROTEINS REGULATING THE CYTOSKELETON AND MEMBRANES IN EV SHEDDING PROCESS

The biogenesis of plasma membrane‐derived vesicles is still poorly understood but it is clear that it requires changes in membrane lipid composition and cytoskeletal regulation (Van Niel et al., 2018). All plasma membrane protrusions are dependent on polymerization of actin and its assisting proteins. Because of its crucial role in protrusion growth and maintenance, actin and its assistant proteins are apparently involved in protrusion‐derived EV shedding process. The role of actin in EV shedding has been shown by inhibition of actin polymerization (by cytochalasin D), which stimulates formation of matrix vesicles in osteoblast‐like cells (Thouverey et al., 2009) and chondrocytes (Hale & Wuthier, 1987). Reorganization of actin cytoskeleton is associated with megakaryocyte‐derived EV formation from tips of protrusions (Flaumenhaft et al., 2009). Also EV shedding from placental syncytiotrophoblast microvilli is associated with actin rearrangements (Han et al., 2016). Activation of platelets by shear stress leads to loss of membrane integrity and disruption of the cytoskeleton, inducing formation of protrusions and microparticles (Tersteeg et al., 2014). Local increase in intracellular pressure or decrease in extracellular pressure (Charras, 2008) causes the membrane separation from the cytoskeleton. According to these findings, the regulatory role of cytoskeleton and its interactions with the plasma membrane during EV formation is obvious.

Interestingly, cryo‐TEM has revealed tubule‐shaped EVs containing actin filaments in human ejaculates (Höög & Lötvall, 2015). Similar EV were detected from human mast cell line HMC‐1 with high number of filopodia (Zabeo et al., 2017). This raises an idea of utilizing actin as a specific marker for protrusion derived EVs. However, the presence of actin cannot be used as a reliable marker for protrusion derived EVs. There is a bulbous structure at the distal tips of enterocyte microvilli (with mean diameter around 100 nm) that does not contain actin filaments (Mcconnell et al., 2009). Similar bulbous extensions were detected at the distal tips of filopodia of HAS3 overexpressing tumour cells with enhanced EV shedding (Rilla et al., 2014). EVs derived from these tip structures may contain only minute amounts or no actin at all. Interestingly, distal tips of actin‐based extensions are typically enriched by functional transmembrane proteins, such as integrins that mediate adhesion of filopodia and thereby promote filopodial extension (Zhang et al., 2004). The possible role of these proteins in disruption and shedding of distal tips as EVs remains to be elucidated.

Earlier studies have demonstrated that EV shedding in tumour cells is regulated by small GTP‐binding protein ARF6 that remodulates actin cytoskeleton (Muralidharan‐Chari et al., 2009). On the contrary, Rac1 activity antagonizes vesicle shedding, while RhoA/ROCK regulated actomyosin‐based contraction promotes EV shedding from tumour cells (Sedgwick et al., 2015) and RhoB/ROCK signaling mediates EV shedding from placental syncytiotrophoblasts (Han et al., 2016). Despite of the growing evidence on actomyosin system in the regulation of EV shedding, more details need to be established to gain a comprehensive understanding on the role of cytoskeleton in this process.

In addition to the cytoskeletal machinery, the formation of EVs from protrusions is controlled by molecules regulating lipid membranes. Prominin‐1 (CD133) is a stem cell marker which associates with cholesterol and is preferentially localized in highly curved plasma membrane protrusions such as microvilli and is carried by EVs of epithelial cells (Marzesco et al., 2005). Cholesterol depletion using methyl‐β‐cyclodextrin resulted in a dramatic change in the microvillar ultrastructure of colon carcinoma cells from a tubular shape to a pearling state and a marked increase in the release of EVs from microvilli (Marzesco et al., 2009). Another example are BAR domain superfamily proteins that are important regulators membrane curvature: N‐BAR and F‐BAR bind to the membrane to form invaginations, such as caveolae and I‐BAR proteins deform the membrane to generate protrusions, such as filopodia (Safari & Suetsugu, 2012). It was suggested that N‐BAR protein endophilin can create points of friction at sites of inwards membrane tubulation via a so‐called friction‐driven scission model (Simunovic et al., 2017). Furthermore, a recent study suggests that external forces promote scission of filopodia with the assistance of I‐BAR proteins to generate filopodia‐derived vesicles (Nishimura et al., 2021). This is an interesting finding to show the role of friction in EV release and to open novel research lines for protrusion‐derived EVs.

## 5 GLYCOCALYX AND ITS ROLE IN EV SHEDDING FROM PROTRUSIONS

The extension and retraction of filopodia are regulated in response to extracellular cues and microenvironmental factors. The glycocalyx is a complex and dynamic system composed of glycolipids, glycoproteins, and polysaccharides anchored to the extracellular leaflet of the plasma membrane of all cells (Varki and Gagneux, 2017). In addition to the intracellular cytoskeleton and lipid plasma membrane, glycocalyx regulates cell shape from the outside to produce filopodia and shedding vesicles (Godula, 2019). Though the mechanisms are still partly unclear, a recent study has shown that glycocalyx can induce plasma membrane instabilities to generate pearled membrane structures and drive the secretion of EVs (Shurer et al., 2019). This study suggests that crowding of glycopolymers generates close contact between adjacent polymer chains and increases unfavourable interactions. This forces the polymers into an extended brush, and the entropic pressures generated by the bound polymer brush can drive the bending of the cell membrane and actin disassembly.

The large, sugar‐rich molecules forming the glycocalyx, called mucins, can extend even hundreds of nanometres above the cell surface. Elevated expression of cell‐surface mucins correlates with enhanced aggressiveness and metastatic potential of the many tumours (Hollingsworth and Swanson, 2004). Furthermore, a typical feature of tumour cells is a high incidence of filopodial extensions on their membranes (Jacquemet et al., 2015) and enhanced secretion of EVs (Xu et al., 2018), which suggests a connection between glycocalyx and these membrane‐derived structures. There is evidence that mucin‐driven formation of protrusions in rat growth plate cartilage is supported by proteoglycans associated with chondrocyte cytoplasmic processes and matrix vesicles (Takagi et al., 1989). Also, cilia of tracheobronchial epithelium were labelled MUC1‐positive (Kesimer et al., 2009). The role of mucins in EV shedding was recently demonstrated in more detail by using native and synthetic mucin polymers, which were able to induce membrane extensions of different numbers and morphology, depending on the cell‐surface concentration of the polymers (Shurer et al., 2019).

Many recent findings suggest that pericellular and extracellular glycosaminoglycans and proteoglycans have a specific role in regulating protrusion formation. Hyaluronan (HA), the most abundant glycosaminoglycan of the glycocalyx, is a strong regulator of plasma membrane curvature. It facilitates the growth of filopodia (Koistinen et al., 2015) and enhances EV budding from their tips (Rilla et al., 2013, 2014). Despite the mechanisms still require elucidation, the most likely explanation is due to ability of HA to increase osmotic pressure. High levels of HA on or near the plasma membrane increase local hydrostatic pressure (Cowman et al., 2015) and give high energy for plasma membrane shaping (Richter et al., 2018). This energy creates a pulling force to modulate the intracellular actin‐myosin‐based contractile cytoskeletal machinery (Shurer et al., 2019). Interestingly, actin withdrawal from the cell periphery results in cell surface blebbing and formation of vesicles at the tips of chondrocyte plasma membrane protrusions (Hale & Wuthier, 1987). As discussed above, actin is not present in the bulbous tips of HAS‐induced filopodia (Koistinen et al., 2015). This may offer a mechanism for enhanced shedding of EVs by HA and may partly explain the ability of HA to induce vesiculation of filopodia to form EVs.

**FIGURE 5:**
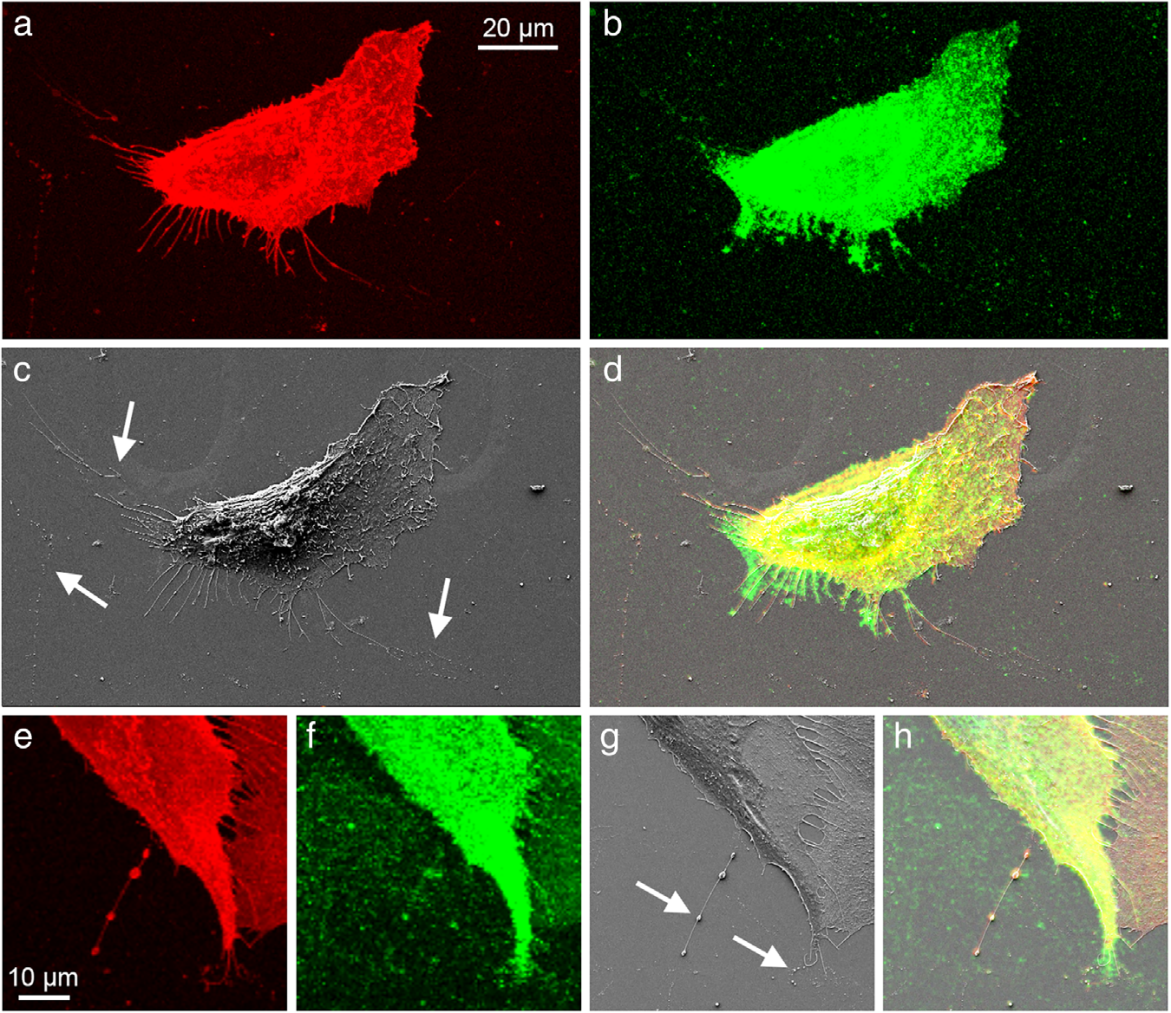
Correlative light and electron microscopy (CLEM) of cultured bone marrow‐derived human mesenchymal stem cells. The cells have numerous CD44‐positive filopodia (a, red) that have a hyaluronan coating (b, green). SEM image is shown in c and overlay is shown in d. Panels e‐h show higher magnification of a pearling protrusion, positive for CD44 (red) and hyaluronan (green). Arrows point EV shedding from retraction fibres (arrows in panel c) and pearling or vesiculation of protrusions (arrows in panel g)

EVs adopt their parental cell's glycocalyx while shedding from the cell's plasma membrane. Recent studies suggest, that EVs budding from filopodia have a glycocalyx that can be detected by both light microscopic (Rilla et al., 2013) and cryo‐TEM techniques (Noble et al., 2020). Interestingly, the glycocalyx could be utilized as a marker to distinguish microvesicles from exosomes and as a specific signature to identify microvesicles derived from plasma membranes (Shurer et al., 2019). All these recent findings suggest that glycocalyx is a substantial inducer of EV shedding and HA‐coated EVs are a unique subgroup of EV with widespread biological relevance (Figure 5).

## 6 MECHANICAL AND CHEMICAL FACTORS AS INDUCERS OF EV SHEDDING FROM PROTRUSIONS

The generation of plasma membrane‐derived vesicles requires plasma membrane blebbing and subsequent shedding. Plasma membrane blebbing is a common feature of active cells during cell movement, cytokinesis, and cell spreading (Charras, 2008). Initiation of membrane blebbing requires either detachment of the plasma membrane from the actin cortex or localized damage of the actin cytoskeleton (Paluch et al., 2005) or induced by osmotic gradients (Pullarkat et al., 2006). For the formation of a bleb, lipids must flow into the bleb or protrusion through its neck (Charras, 2008). Sanborn et al. have experimentally observed, that negative osmotic gradient results in the influx of water and transient pearling and vesiculation of retracting phospholipid membrane tubes (Sanborn et al., 2013).

**FIGURE 6:**
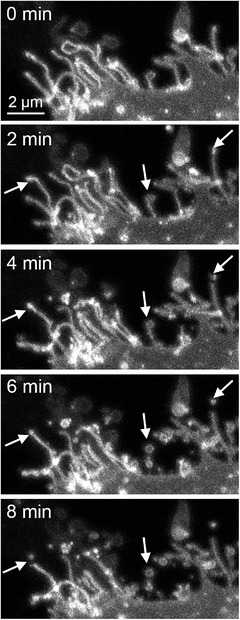
Confocal time‐lapse live‐cell imaging demonstrates the shedding of EVs from the tips of plasma membrane protrusions (arrows). Images in all time points show 3D maximum intensity projections created from stacks of confocal optical sections. One edge of a live MCF7 breast cancer cell is shown

In addition to their various cellular functions, filopodia are suggested to act as osmo‐sensors, which protect the cells against osmotic stress (Karlsson et al., 2013). However, the slender plasma membrane structures are susceptible to different mechanical (shear stress), stressful and physical (light and radiation), chemical (osmotic changes), and environmental factors (pH, acidic environment). Aquaporins are transmembrane water channels that promote plasma membrane protrusions by increasing the water flux and weakening membrane‐cytoskeleton interactions. Karlsson et al. have shown that the addition of water, resulting in a local reduction in osmotic pressure, induces blebbing of these protrusions (Karlsson et al., 2013). However, in many cases, the plasma membrane blebbing is followed by retraction (Charras, 2008), without actual detachment of the bleb from the cell body. Thus, live‐cell imaging experiments are the most reliable way to demonstrate the actual detachment of EVs from the plasma membrane of the parental cells (Figure 6). However, during live cell imaging experiments, phototoxicity induced by both the low‐ and high‐wavelength light that is used to illuminate the cells may induce unwanted and artefactual blebbing (Kiepas et al., 2020).

Many epithelial cells are subjected to shear stress and plasma membrane blebbing can be locally controlled by shear stress (Albrecht‐Buehler, 1980). Shear stress is the mechanical force that is induced by the friction of liquid against the apical plasma membrane. In vivo, several adherent cell types, such as endothelial cells lining blood and lymphatic vessels or nephrons, but also cancer cells (Kang et al., 2018), are continuously exposed to mechanical forces such as fluid shear stress, stretching, and hydrostatic pressure (Gordon et al., 2020). These forces are generated mainly by blood and interstitial flows and influence the cell morphology, physiological behaviour, and adhesion properties of cells in many ways, but they also potentially induce shedding of EVs, especially on cells with thin and fragile protrusions. One example of the effect of shear stress is from platelets: long membrane protrusions induced by physiological flow in platelets lead to the formation of microparticles (Tersteeg et al., 2014). The pericellular glycocalyx discussed above is an important structure in the mechanotransduction of interstitial flow‐induced shear stress (Kang et al., 2018). Therefore, it may be associated also with shear stress‐induced shedding of EVs.

Interestingly, diverse EV groups, including long EV with inside cytoskeletal structures have been detected in human sperm by cryotechniques (Höög & Lötvall, 2015), which suggests that a population of EV may originate from the protrusions of epithelial cells lining the urogenital tract. The cells lining the epithelia of the prostate gland, seminal vesicle, and vas deferens have typically long plasma membrane extensions, and shear stress may induce shedding of tips or even bigger sections of microvilli and other protrusions expressed in the linings of these cavities.

Many other examples of chemical or mechanical factors that may induce EV shedding have been observed. Plasma membrane disruption resulting from mechanical wounding is known to induce exocytosis and the formation of microvilli (Miyake & McNeil, 1995). Danger signals such as lipopolysaccharide induce filopodia and EV formation in human mesenchymal stem cells (Arasu et al., 2017) and enhance EV secretion of dendritic cells (Obregon et al., 2006). Oxygen‐glucose deprivation conditions typical for preeclampsia induce EV shedding from placental syncytiotrophoblasts (Han et al., 2016).

## 7 ASSOCIATION OF PROTRUSION DERIVED EVS WITH CELL MIGRATION

The invasive cancer cells interact very closely with their microenvironment, and protrusions such as filopodia are essential for their adhesion and invasion. Migrating cells use filopodia to probe their environment, and maturation of filopodial tip adhesions to focal adhesions is known to direct cell migration (Jacquemet et al., 2015, 2019). This suggests that migrating cells potentially secrete more actively EVs than non‐moving cells. Actually Sung et al have shown that directional cell migration is dependent on EV secretion (Sung et al., 2015). There is also evidence that ‘footprints’ originate from retraction fibres or fractionation of cellular protrusions of human mesenchymal stem cells (Arasu et al., 2017). Correlative light and electron microscopy (Figure 5) has been a versatile method to reliably detect EV from retraction fibres or vesiculation of protrusions as ‘footprints’, or ‘adhesive exosome trails’ (Arasu et al., 2017; Sung et al., 2020). In a recent live‐cell imaging study, CD44‐ and integrin αVβ5‐positive EVs were shown to be arranged in ‘trails’, which are likely derived from cleavage of filopodial tips of trabecular meshwork cells, as they retract to the cell body (Keller & Kopczynski, 2020).

One of the interesting subgroups of large EVs (up to half of the area of their parent cells, diameter up to several micrometres) are so‐called microplasts, which are membrane bound cytoplasmic fragments capable of autonomous movements. Microplasts, that could be categorized as ectosomes, were first observed as early as in 1980s in skin fibroblasts (Albrecht‐Buehler, 1980) and later in glioma cells (Yount et al., 2007). Many similarities with microplasts can be found from EVs described by Johnson et al., which contain an active cytoskeleton and are independently capable of dynamic shape‐changes and even active motility (Johnson et al., 2017). Additionally, recent data suggest that microplasts originate from tunnelling nanotubes (TNTs) of MCF7 cells upon induction by macrophage‐conditioned medium (Patheja & Sahu, 2017). This may be related to a recent report which demonstrated that rolling neutrophils on the vessel wall form tethers that, when detached, form elongated neutrophil‐derived structures (ENDS). These novel types of neutrophil‐derived microparticles do not express tetraspanins but represent another specific type of EVs originating from cellular extensions (Marki et al., 2021).

One more migration‐related species of EVs are migrasomes (up to 3000 nm), oval‐shaped vesicular organelles that are formed during cell migration from tips of retraction fibres behind migrating cells (Figure 3). They are believed to act as organelles that mediate the release of cytoplasmic contents during cell migration (Ma et al., 2015). They express tetraspanin‐4 as a specific marker, which is known to interact with EV markers CD81, CD9 and integrins (Tachibana et al., 1997). Interestingly, tetraspanin‐4 and 7 are required for migrasome formation (Jiang et al., 2019). They are typically large vesicles, which contain numerous smaller vesicles, grow on the tips of retraction fibres that eventually break and release the vesicles into the extracellular space. Despite their detailed function has not been so far elucidated, there is evidence that their release is dependent on cell migration (Ma et al., 2015) and they are associated with organ morphogenesis during zebrafish gastrulation (Jiang et al., 2019).

## 8 FILOPODIA AS SPECIFIC PLATFORMS FOR EV TARGETING AND UPTAKE

This article is mainly focused on the role of extensions as platforms for EV release, but in some cases filopodia may also enhance uptake of EVs into their target cells. The study of (Heusermann et al., 2016) suggests a role of filopodia in trapping and subsequent internalization of EVs. In the study of Thayanithy et al., EVs induced tunnelling nanotube formation in mesothelioma cells and utilized those nanotubes as potential uptake and cell‐to‐cell transport of themselves (Thayanithy et al., 2014). Recent studies by Franchi et al. showed that tunnelling nanotubes (TNTs) of MDA‐MB‐231 breast cancer cells are associated with EVs surfing from one cell to another (Franchi et al., 2020a, 2020b). Interestingly, a recent study by Wen et al demonstrated that coronaviruses surf along filopodia on the host membrane to the entry sites (Wen 2020), suggesting that cellular entry of viruses may share similar features with the entry of EVs.

## 9 FINAL CONCLUSIONS

It can be summarized that the secretion dynamics of EVs from plasma membrane protrusions reflect the microenvironmental changes, parent cell activity, or stress, and is regulated by the plasticity of the plasma membrane and dynamic activity of cytoskeleton, glycocalyx, shear stress and osmotic changes and other mechanical, chemical and molecular factors. During this process, loss of asymmetrical distribution of phospholipids and cytoskeletal reorganization are the putative factors that favour membrane budding (Morel et al., 2011), and increase the probability of formation and shedding of EVs.

**FIGURE 7:**
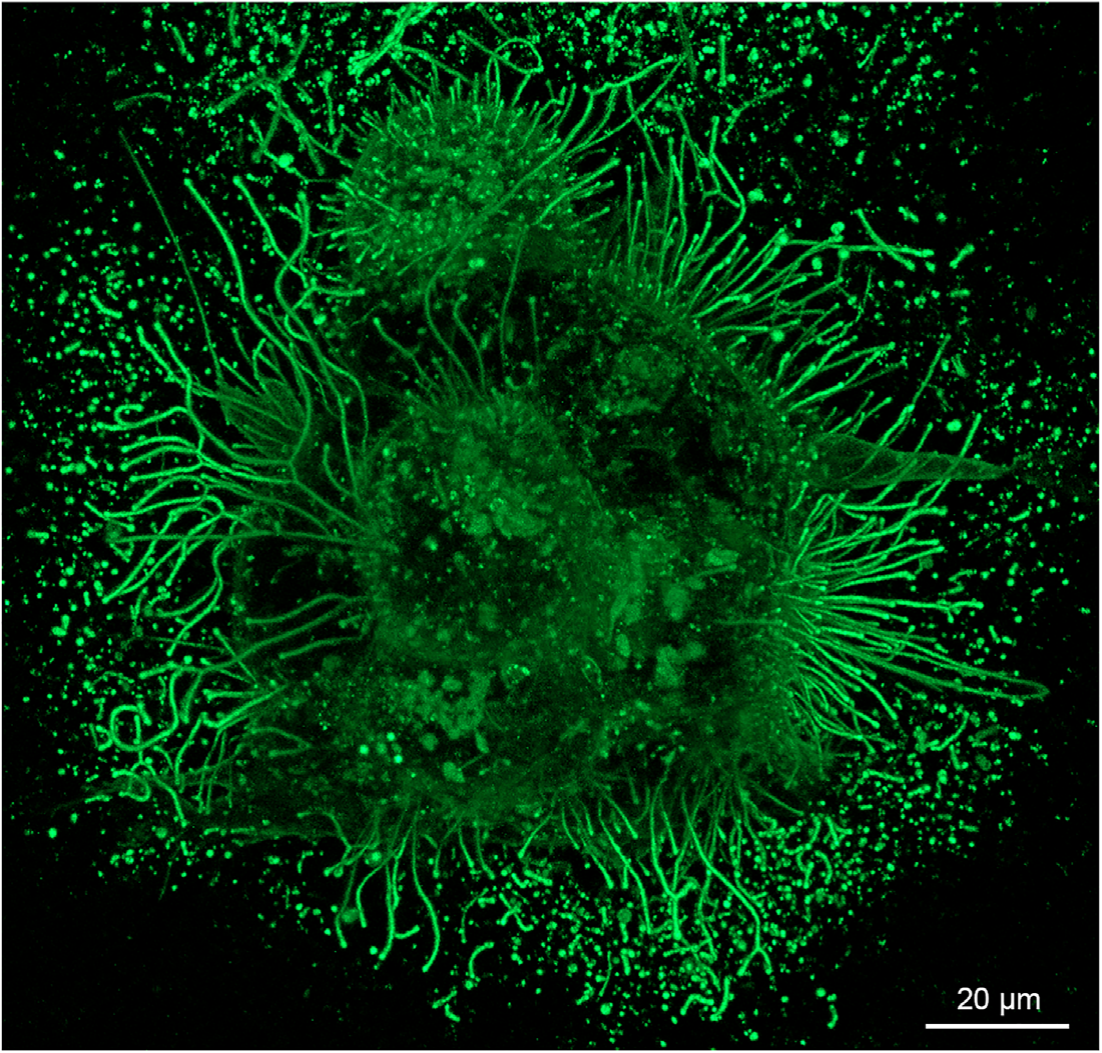
A sphere of live GFP‐HAS3 expressing MCF‐7 breast cancer cells grown in 3D collagen gel. HAS3 expression induces the formation of extremely long filopodia and secretion of EVs from their tips. EVs are visualized in high numbers because they are trapped inside the gel. The image shows a maximum intensity projection created from a stack of confocal optical sections

What are the main advantages for a single cell of creating EVs from membrane extensions instead of the main cell body? The higher the plasma membrane curvature is, the less energy is needed for EV shedding (Shurer et al., 2019), suggesting that plasma membrane fragment blebbing and shedding from the tips of filopodia are energetically more favourable as compared to the plain plasma membrane (Richter et al., 2018). Furthermore, after EV release, the local environment and tissue architecture impacts the rate of diffusion of EVs towards target cells or tissues. EV release from long protrusions, extending far from the donor cell body, potentially promotes the efficiency of EV distribution and spreading in the cellular microenvironment. This is particularly important in tissues where cells are embedded in a mechanically dense environment such as in bone and cartilage or in tumour matrix. Unfortunately, the majority of current in vitro EV studies are performed in two‐dimensional monolayer cell cultures, where EVs are secreted directly into the culture medium and lack the interactions with the ECM. This creates a huge gap in the current knowledge about EV shedding mechanisms and regulation. A sphere of live human breast cancer cells in collagen gel with GFP‐HAS3‐induced extensions and EVs indicate the activity of this process also in 3D environment (Figure 7). To understand these processes, it is highly important to perform the future studies in 3D conditions.

A challenging question is how to identify and quantify the proportion of extension‐derived EVs from other EV subpopulations? EV originating from outer plasma membrane structures are potentially larger in diameter than exosomes generated by exocytosis from the intracellular compartment. However, the typical diameter of filopodia is 130 nm (Kultti et al., 2006), suggesting that the diameter of EV originating from their tips overlaps with the size of exosomes (50 ‐ 200 nm). Thus, it will be challenging to utilize differences in diameter to identify protrusion‐derived EV from EV preparations that contain also EVs generated by exocytosis or other mechanisms. Another challenge is, whether the different molecular compositions could be utilized for the identification of extension‐derived EVs. One of the most promising markers could be actin or other molecules associated with the cytoskeletal machinery of protrusions. However, as discussed before, the tip structures of microvilli and filopodia contain only minute amounts of actin, which decreases the reliability of actin as a specific marker. Interestingly, of the most common EV markers, tetraspanins CD9 and CD81 are localized on filopodia, while CD63 is mostly expressed in the intracellular compartment (Peñas et al., 2000). Another promising marker is the microvillar protein prominin‐1 (CD133), that is not expressed in vesicles carrying CD63 typical for exosomes (Marzesco et al., 2005) However, the level of specificity of tetraspanins as markers for extension‐derived EVs remains to be confirmed. A recent study by Nishimura et al. describes the specific proteins (IRS4 and Rac1) carried by filopodia‐derived EVs and their enhanced effect on the migration of target cells (Nishimura et al., 2021). These findings are promising for more specific identification and determination of the proportion of the EVs originated from plasma membrane protrusions.

There is growing evidence that EV shedding from diverse plasma membrane protrusions is a general process, sharing many common mechanisms related to the regulation of cytoskeleton and lipid membranes. However, the diversity of protrusions in morphology and size, molecular composition, function and dynamics complicates the characterization of EVs derived from them and suggests that diverse mechanisms account for their formation. But what will be the relevance of recognizing this mechanism of EV generation for research and clinical applications? It is undeniably meaningful for the assembly and regeneration of connective tissues, maintenance of stem cells, functions and homeostasis of different epithelia, and interaction of tumour cells with their microenvironment. A deeper understanding of the EV shedding mechanisms helps to develop efficient and specific EV‐based biomedical applications, such as drug loading and controlled delivery. Furthermore, the EVs originating from cellular extensions may reflect the changes in cellular morphology, metabolic stage, and invasion potential, offering novel insights for diagnostic purposes.

## CONFLICT OF INTERESTS

There is no conflict of interest regarding the publication of this article.
